# Exploratory study of the effect of DHA supplementation on blood fatty acids and inflammatory markers in children with MIS-C

**DOI:** 10.3389/fnut.2025.1597868

**Published:** 2025-07-16

**Authors:** Elvira Verduci, Patrizia Risè, Giulia Fiore, Sara Vizzuso, Alice Bonomi, Dario Dilillo, Laura Fiori, Elisabetta Di Profio, Valeria Calcaterra, Savina Mannarino, Elena Zoia, Enza D’Auria, Angelo Sala, Gianvincenzo Zuccotti

**Affiliations:** ^1^Department of Health Sciences, University of Milan, Milan, Italy; ^2^Metabolic Diseases Unit, Department of Pediatrics, Vittore Buzzi Children’s Hospital, University of Milan, Milan, Italy; ^3^Department of Pharmaceutical Sciences, University of Milan, Milan, Italy; ^4^Department of Pediatrics, Vittore Buzzi Children’s Hospital, University of Milan, Milan, Italy; ^5^Monzino Cardiology Centre IRCCS, Milan, Italy; ^6^Pediatric Cardiology Unit, Vittore Buzzi Children’s Hospital, Milan, Italy; ^7^Anesthesia and Intensive Care Unit, Vittore Buzzi Children’s Hospital, Milan, Italy; ^8^Allergology and Pneumology Unit, Vittore Buzzi Children’s Hospital, Milan, Italy; ^9^Department of Biomedical and Clinical Sciences, University of Milan, Milan, Italy

**Keywords:** SARS-CoV-2, long COVID, hospitalized children, polyunsaturated fatty acids, docosahexaenoic acid, inflammation

## Abstract

**Background and aims:**

Children infected with SARS-CoV-2 may develop multisystem inflammatory syndrome (MIS-C) 4–6 weeks after exposure. MIS-C is characterized by elevated markers of inflammation and low blood values of linoleic acid (LA), arachidonic acid (AA) and docosahexaenoic acid (DHA) during acute phase. The aim of this pilot exploratory study was to assess the short-term beneficial impact on the blood fatty acid profile following DHA supplementation in children who have suffered from MIS-C.

**Methods:**

Fifty-two children aged 2–18 years with diagnosed MIS-C, were enrolled between December ‘20 and March ‘22. Blood samples were collected at hospital discharge (T0), and at 3 (T1) and 6 months (T2) post-discharge using dried blood spots for fatty acid analysis by gas chromatography. Inflammatory and metabolic blood markers were assessed at T0 and T2. All participants received healthy dietary advice throughout the study. In Group 1 23 consecutive patients received DHA supplementation (250 mg/day of DHA) from T0 to T1, followed by dietary advice alone until T2. In Group 2 29 children with MIS-C received only dietary advice throughout the observation period.

**Results:**

An altered inflammatory status, independent of treatment, was shown in all children compared to pediatric reference values. After intervention, Group 1 experienced a significant enrichment in both total n-6 and n-3 blood FAs when compared to baseline (*p* < 0.0001). Specifically, there was a significant increase of DHA (1.19 ± 0.25 at T0 vs. 2.67 ± 0.78 at T1) and EPA (0.32 ± 0.09 at T0 vs. 0.46 ± 0.10 at T1) levels, that remained consistent at T2 (*p* = 0.0002 and *p* < 0.0001, respectively). Within Group 2 only n-3 alpha linolenic acid (ALA) significantly increased at T1 compared to baseline (*p* < 0.05). The total increase in n-3 after intervention (ΔT1-T0) was significantly higher in Group 1 compared to Group 2 [1.90(0.9) vs. 0.49(0.8), *p* < 0.0001 and *p*_adj_ = 0.005]. Erythrocyte sedimentation rate (ESR) and IL-6 showed a better tendency toward normalization in Group 1, although without statistical significance.

**Conclusion:**

This pilot study is the first to explore the potential effects of DHA supplementation in children with MIS-C. DHA was associated with improvements in the blood fatty acid profile, which persisted beyond the supplementation period, and showed a trend toward normalization of selected biochemical parameters. Further adequately powered, controlled studies are needed to confirm these observations and to evaluate the potential role of early n-3 PUFA supplementation during the stable and recovery phases in critically ill pediatric patients.

## 1 Introduction

Children infected with SARS-CoV-2 may develop multisystem inflammatory syndrome (MIS-C) 4–6 weeks after exposure (1). MIS-C occurs with an incidence of approximately 1 out of 3,000 to 4,000 children and adolescents with documented COVID-19 infection (2–4), and the demographic characteristics of the patients showed a male predominance and an age range between 7 and 10 years (5, 6). Multiple organ failure is a consequence of the disease, manifested by gastrointestinal, cardiovascular, hematological, mucocutaneous, neurological, and respiratory symptoms (7). Intensive care unit (ICU) care is required for patients with more severe disease (8), and according to several studies, it may occur in 50–80% of children with MIS–C (9). A primary role of a cytokine storm and the impact of adaptive immunity following SARS-CoV-2 infection are described in the most widely accepted explanation of the disease mechanism of MIS-C (10, 11). The pathophysiology of MIS-C involves complex inflammatory cascades similar to those observed in severe COVID-19, characterized by dysregulated immune responses and altered lipid metabolism (12, 13). Recent studies have identified unique neutrophil gene signatures associated with COVID-19 severity progression (14), suggesting that inflammatory biomarkers may serve as prognostic indicators for post-COVID-19 syndromes including MIS-C. Additionally, some intrinsic susceptibility factors have been described, and evidence of molecular mimicry for MIS-C pathogenesis has also been provided (15). During the acute phase of MIS-C, altered glucose metabolism (elevated HOmeostasis Model Assessment for Insulin Resistance (HOMA-IR) and Triglyceride Index values) and a reduction in body mass index (BMI) z-score have been reported in children (16, 17).

COVID-19 and its sequelae, including MIS-C, are characterized by profound alterations in lipid metabolism and inflammatory mediator production. Studies have demonstrated that SARS-CoV-2 infection leads to disturbances in fatty acid (FA) biosynthesis pathways and increased plasma arachidonic acid (AA) levels (18), while also affecting sphingolipid metabolism, particularly sphingomyelin levels, which correlate with disease severity (13). Additionally, the inflammatory response involves matrix metalloproteinase activation and lipid peroxidation processes that contribute to tissue damage (19). Previously we reported lower levels of omega-6 (ω6) linoleic acid (LA) and AA in MIS-C children, probably as result of massive release from phospholipid stores followed by metabolic conversion into pro-inflammatory lipid mediators (20). In particular, during the acute phase of MIS-C in children, blood levels of LA and AA were lower by an average of 38 and 35%, respectively (20), when compared to previously reported values for the pediatric population (21–25). Omega-3 fatty acids have also been strongly investigated in relation to immune status and inflammatory processes, particularly the anti-inflammatory and pro-resolving properties of metabolites derived from omega-3 polyunsaturated fatty acids (ω3 PUFA). In fact, metabolites synthesized from docosahexaenoic acid (DHA) and eicosapentaenoic acid (EPA) by means of lipoxygenases’ activity, have been collectively defined as specialized pro-resolving mediators (SPMs) (26). Hence, it is not surprisingly that we previously observed lower plasma levels of DHA in children with acute MIS-C compared to controls (20). SPMs are compounds involved in tissue regeneration and includes resolvins, protectins, maresins, and maresins conjugate (27). By activating macrophages with an anti-inflammatory phenotype (M2) and promoting phagocytosis in a non-phlogistic manner, SPMs produced by the metabolism of ω3 PUFA have been shown to reduce pro-inflammatory mediators’ synthesis and neutrophil recruitment (20).

In adult patients higher circulating ω3 PUFA, have been associated with a better prognosis for COVID-19 (28). Harris et al. investigated the levels of blood DHA in a large, prospective, population-based cohort of 110,584 individuals to compare the risk of COVID-19 outcomes including testing positive for SARS-CoV-2, hospitalization, and death, in relation to the baseline plasma DHA levels. The findings indicate that individuals in the highest quintile of plasma levels of DHA, experience a 26% reduced risk of hospitalization, positive test outcomes, and mortality, compared to those in the lowest (29). A limited number of studies also aimed at assessing the omega-3 Index (O3I), a measure of the EPA and DHA content (as %) of red blood cells, usually expressed as a proportion of the total weight of fatty acids in red blood cell membranes (30). Zapata et al. (31) in their cross-sectional study confirmed previous findings, revealing that patients with severe COVID-19 had a reduced O3I, consistent with insufficient fish and ω3 supplement consumption, and markedly lower than the healthy control subjects. On the other hand, the higher the O3I and the lower was the risk of requiring mechanical ventilation and mortality (32). Overall, the identification of reliable biomarkers for COVID-19-related inflammatory syndromes has become crucial for patient management. Sphingomyelin species have emerged as potential prognostic biomarkers, with elevated levels correlating with age, hospitalization duration, and inflammatory markers such as interleukin-6 (IL-6) and interleukin-8 (13). Furthermore, specific neutrophil gene signatures have been associated with the turning point from non-severe to severe COVID-19 forms (14).

Given the central role of lipid mediators in COVID-19 pathophysiology, nutritional interventions targeting these pathways represent promising therapeutic approaches. The interplay between glucocorticoid therapy and lipid mediator pathways influences inflammatory responses in COVID-19 (12). Specifically, regarding ω3 supplements, a systematic review in adult patients, estimated them to be 12 to 21% effective in reducing the risk of Covid-19 (33). Additionally, a scoping review of adult patients highlights their potential role in treatment (34). Specifically, one randomized controlled trial (RCT) examined the effects of enteral ω3 supplementation (1,000 mg daily containing 400 mg EPA and 200 mg DHA) for 14 days in critically ill COVID-19 patients. The treated group showed higher one-month survival rate, renal and respiratory function, compared to controls (35). The latter is supported also by several studies reporting that ω3 FA can reduce mortality rates in critically ill patients (36, 37).

To date there are no studies that have evaluated the effects of supplementation with ω3 FA in children affected by SARS-CoV-2.

The present study aims for the first time to assess the short-term beneficial impact on the blood fatty acid profile following DHA supplementation in children who have suffered from MIS-C.

## 2 Materials and methods

### 2.1 Subjects

In this exploratory pilot study, 52 children and adolescents aged 2–18 years with MIS-C, defined according to the Centers for Disease Control and Prevention (CDC) classification (2), were recruited between 1 December 2020 and the end of March 2022 at the Pediatric Department of Children’s Hospital Vittore Buzzi in Milan, Italy.

Multidisciplinary management in the acute phase was previously described (15). For each children pediatric intensive care unit (PICU) admission and the overall number of days of hospitalization were recorded during the progression of the diseases, as previously described (38). During hospital stay, standard drug therapy was administered to all patients (intravenous immunoglobulin, corticosteroids, and antiplatelet therapy).

Patients were enrolled after 5–7 days from admission in Pediatric Unit during the stable phase of the disease. Upon admission, for all patients, a clinical and biochemical evaluation was conducted, and anthropometric measurements were taken at admission and before hospital discharge. During hospital stay (T0) a blood droplet was collected from each patient over a special adsorbent paper embedded with the antioxidant butylated hydroxytoluene (BHT) and stored in a refrigerator until analysis, as described below. Additionally, the drug therapy of the children was recorded at this time. After discharge, the following timepoints of the study were scheduled: T1 90 days after T0, and T2 180 days after T0.

**Figure 1:**
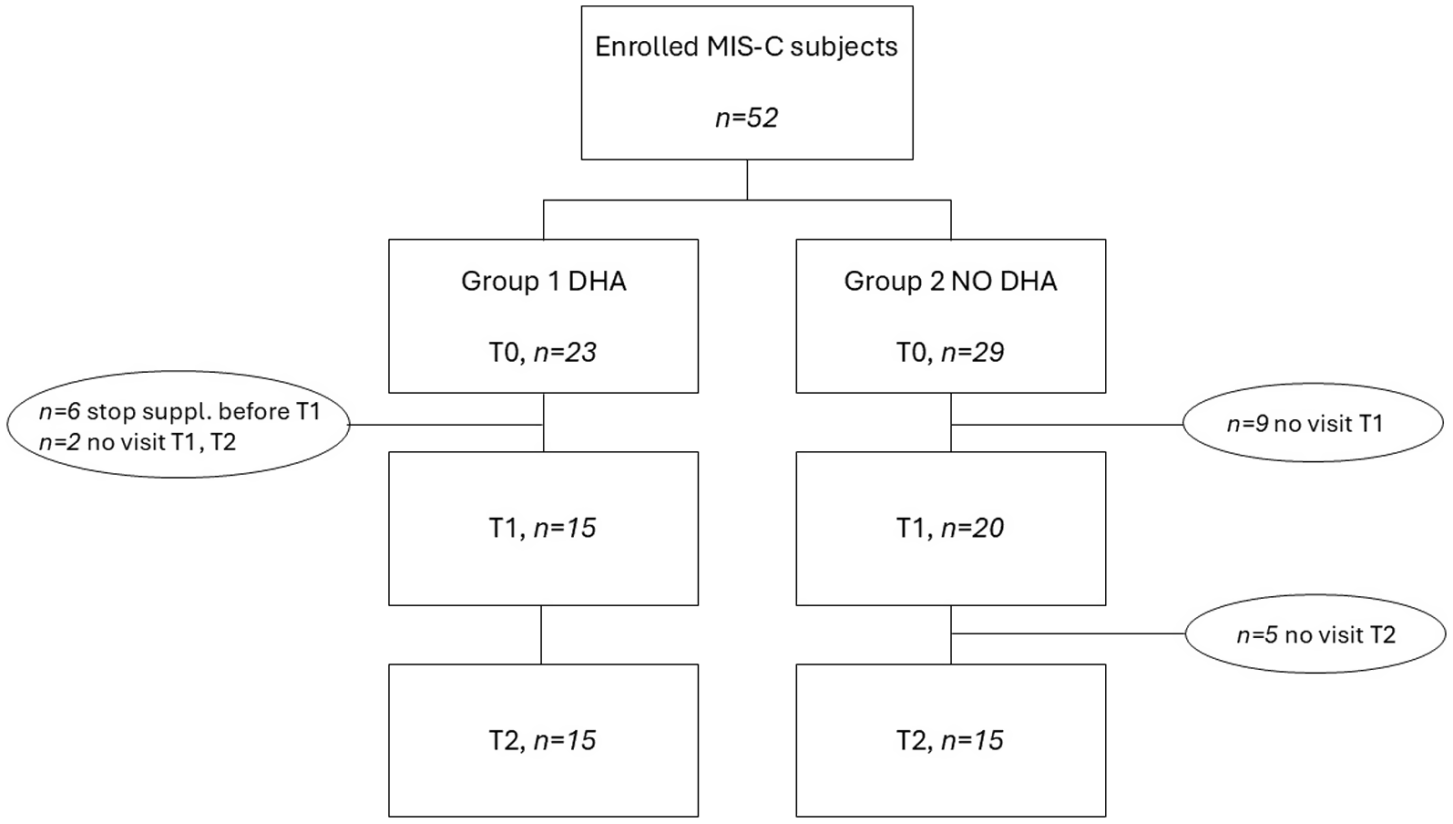
Flow chart of enrolled patients.

Dietary advice on healthy diet, in accordance with the principles of the Mediterranean diet, was provided to every participant from T0 up until the end of the study period (T2). From T0 to T1, enrolled patients were assigned to one group of study intervention. At discharge (T0), 23 consecutive patients received instructions for DHA supplementation (1 mL/day equals to 250 mg of DHA; see Supplementary Table 1) for 3 consecutive months (Group 1). Meanwhile, 29 children with MIS-C, at T0 did not receive any DHA supplementation (see Figure 1) but only usual care with dietary advice (Group 2). Both groups of intervention were asked not to take any additional supplement during the entire study period. A monthly telephone call was carried out to check adherence to supplementation and dietary advice. At T1 and T2, other samples on BHT absorbent paper were taken and stored in accordance with the previously mentioned procedure. Moreover, at T2, the patients underwent further multidisciplinary examinations, clinical and biochemical analysis were performed.

The study complies with the Declaration of Helsinki guidelines and was approved by the hospital’s Institutional Review Board (protocol number 2021/ST/004). After being informed about the study, each caregivers provided written consent for inclusion in the study.

### 2.2 Study product

At discharge children in Group 1 were instructed to take 1 mL/day of DHA oil supplement, equals to 250 mg of DHA from algae origin. The rationale for DHA supplementation was based on evidence demonstrating the role of lipid mediators in COVID-19 pathophysiology, including platelet-activating factor and endocannabinoid pathways (12), and the potential for omega-3 fatty acids to counteract oxidative stress and matrix metalloproteinase-mediated tissue damage (19). Details on nutritional composition of the study product are reported in Supplementary Table 1. The rationale for using 250 md/day was derived from previous literature, as we considered both the established condition of insulin resistance observed in our cohort of pediatric patients with MIS-C (16) and the minimum observed effect of dosage of DHA reported in the literature among pediatric age groups (39).

### 2.3 Anthropometry and blood parameters

At T0, physical examination was performed and anthropometric measurements of weight and height were taken, and the BMI was calculated for each patient. A mechanical column scale with altimeter (Seca 711 and Seca 220) was used to measure weight and height, while a tape measure (Seca 201) was used to measure arm and waist circumferences. Tricipital skinfolds were also measured using a caliper (Holtain 610). BMI (kg/m^2^) and BMI z-score were calculated according to CDC growth chart reference values (40, 41). The diagnosis of MIS-C was confirmed by performing a complete blood count and measuring levels of C-reactive protein (CRP), erythrocyte sedimentation rate (ESR), procalcitonin, ferritin, cardiac troponin T (cTnT), N-terminal pro-brain natriuretic peptide (NT-proBNP), coagulative parameters, creatine kinase, electrolytes, and IL-6. The selection of inflammatory markers (IL-6, CRP, ESR) was based on their established correlation with COVID-19 severity and lipid metabolism alterations. These markers correlate with sphingolipid disturbances (13) and neutrophil activation signatures (14) in COVID-19 patients.

Furthermore, a fasting blood sample was collected between 8:30 and 9:00 a.m. during hospitalization to evaluate the metabolic profile. The following parameters were evaluated: total cholesterol, HDL cholesterol, LDL cholesterol, fasting plasma glucose, and triglycerides (TG). The triglyceride–glucose (TyG) index, a surrogate for insulin resistance, was calculated as [ln(fasting triglycerides (mg/dL) fasting plasma glucose (mg/dL)/2)] (42).

All blood testing were repeated after 6 months at T2, excluding parameters closely linked to MIS-C diagnosis.

### 2.4 Fatty acid analysis

The FA profile was assessed using a blood droplet gathered on a special adsorbent paper embedded with the antioxidant BHT. FA methyl esters were examined by gas chromatography, following direct transmethylation, utilizing a GC-2100 (Shimadzu Italia S.r.l., Milano, Italy) equipped with a 15 m capillary column (DBB Agilent), PTV injector and FID detection (43).

Relative percentages were used to report 23 different FAs; total saturated FA (SFA), monounsaturated FA (MUFA) and PUFA were also reported. In addition, O3I was calculated according to the method outlined by Stark et al. (44). The unsaturation index (U.I.) was calculated as the sum of the percentage levels of each FA x its number of double bonds.

### 2.5 Statistical analysis

Continuous and categorical data are expressed as mean [Standard Deviation (SD)] and number (percentage %), respectively. All variables were tested for normality distribution according to Shapiro Wilk test. Baseline comparisons of anthropometric data, clinical features, and fatty acids profile were tested according to independent t-test for unpaired data or Mann Whitney U Test for normally and not normally distributed variables, respectively. Chi square test was performed for categorical variables at baseline. To account for potential baseline differences between the DHA-supplemented and control groups due to non-randomized, consecutive patient allocation, a propensity score was calculated for each subject using logistic regression. Variables included in the model were sex, PICU admission, baseline inflammatory markers (ferritin, D-dimer, fibrinogen), and relevant FAs (ALA, EPA, Gamma-linolenic acid, and AA). Fatty acids comparisons over time within the same group were performed with ANOVA for repeated measure and Friedman test with Bonferroni correction for normally and not normally distributed variables, respectively. Significant changes over time for each fatty acid within each group were expressed as ΔT1-T0, ΔT2-T0, and ΔT2-T1, and after were compared between Group 1 (suppl DHA) vs. Group 2 (no DHA). In the first model, raw data of delta fatty acids profile between groups were compared with independent t-test for unpaired data or Mann Whitney U Test for normally and not normally distributed variables, respectively. In the second model, propensity scores were used as covariates in adjusted comparisons of outcomes between groups. For clinical biomarkers, changes between T2 and T0 were described as *Δ* and compared between the two groups. Delta of the difference between groups (ΔG2-G1), was expressed as the difference in means with 95% confidence intervals based on the unpaired t-test for normally distributed variables, or as the Hodges–Lehmann estimate of the median difference with 95% confidence intervals based on the Mann–Whitney test for non-normally distributed variable. Effect sizes were estimated according to Cohen’s d or non-parametric r where appropriate. A two-sided *p*-value less than 0.05 was required for statistical significance. The IBM SPSS Statistics 28.01v software and GraphPad Prism 9.0 were used to conduct the statistical analysis.

## 3 Results

Figure 1 shows the flow chart of enrollment according to groups. Overall, 52 subjects (children and adolescents) with MIS-C were enrolled at T0. In the supplemented group (Group 1), 6 children took DHA for less than 3 months and 2 children did not attend the visits at T1 and T2; thus, they were excluded from final analysis. No adverse events were reported during the study and all participants tolerated the DHA supplement well, and no safety concerns were raised by caregivers or investigators. In Group 2, 9 children did not attend the visit at T1, and 5 children were excluded from the final analysis as they did not attend the visit at T2. At 6 months after the acute event (T2) the two groups consisted of 15 subjects each.

Table 1 shows general characteristics, clinical manifestation and anthropometric indices of the study groups at baseline. Apart from a significant difference in sex at baseline, no differences were observed for anthropometric, clinical and biochemical parameters at baseline among groups. Specifically, the length of hospitalization was 13(5.0) and 15(4.0) days in Group 1 and 2, respectively (*p* = 0.15). Rate of PICU admission was not significantly different among groups (67% vs. 40%, respectively, *p* = 0.14). Regarding fatty acid profile at baseline (see Supplementary Table 2), both groups were comparable except for a significantly higher value of gamma-linolenic acid (*p* = 0.014) and AA (*p* = 0.031), and a lower value of EPA (*p* = 0.031) in Group 1 compared to Group 2. There were no significant differences in dietary intakes among the two groups (data not shown). The mean propensity scores were 0.83 ± 0.23 in Group 1 and 0.19 ± 0.27 in Group 2. These scores were included as covariates in all between-group comparisons to adjust for potential confounding.

**Table 1:** Characteristics and anthropometric indices of study groups at baseline (T0).

Variables	Group 1 T0 (DHA)	Group 2 T0 (NO DHA)	*p* value
Total (*n*)	15	15	
Male *n* = 21 (70%) §	13 (87%)	8 (53%)	**0.05**
Age (y)	8.313 (3.96)	9.227 (3.56)	0.52
Length of hospitalization	13 (5.0)	15 (4.0)	0.15
PICU admission^§^	10 (67%)	6 (40%)	0.14
Height	1.299 (0.25)	1.361 (0.24)	0.49
Weight	32.83 (15.77)	33.75 (13.61)	0.86
BMI z score CDC	0.5153 (0.89)	0.2493 (1.11)	0.80
*Biochemistry*			
CRP (mg/L)	199.8 (105.6)	175.5 (88.3)	0.50
Ferritin (μg/L)	1537.1 (1,881)	593.7 (504.2)	0.11
ESR (mm/h)	51 (27.2)	59.9 (32.6)	0.46
IL-6 (ng/L)	31.88 (65.6)	17.94 (17.2)	0.66
Albumin (g/dL)	2.6 (0.4)	2.8 (0.5)	0.28
D-Dimer (μg/L)	3,7635 (3,536)	5736.7 (6,062)	0.30
Fibrinogen (g/L)	6.5 (0.8)	5.6 (1.5)	0.49
Glucose (mg/dL)	111.53 (37.11)	118.36 (30.81)	0.59
Cholesterol total (mg/dL)	129.3 (46.94)	145.1 (43.15)	0.35
HDL (mg/dL)	15.13 (10.60)	23.07 (15.11)	0.11
LDL (mg/dL)	70.25 (31.81)	86.91 (27.87)	0.19
Triglycerides (mg/dL)	227 (96.42)	207.29 (103.47)	0.64
TyG index	9.30 (0.52)	9.28 (0.47)	0.92

BMI z score CDC, Body Mass Index z-score according to Centers for Disease Control and Prevention; CRP, C-Reactive Protein; ESR, Erythrocyte Sedimentation Rate; HDL, High-Density Lipoprotein Cholesterol; IL-6, Interleukin-6; LDL, Low-Density Lipoprotein Cholesterol; PICU, Pediatric Intensive Care Unit; TyG Index, Triglyceride-Glucose Index. *p* value calculated with unpaired t test or Mann Whitney U test for normally and not normally distributed variables, respectively. ^§^*p*-value according to Chi square test. *p*-values in bold indicate statistical significance (*p* < 0.05).

Table 2 shows the FA profile in Group 1 at different timepoints. Compared to baseline, at T1 there was a significant increase of DHA levels (1.19 ± 0.25 at T0 vs. 2.67 ± 0,78 at T1), and also of docosapentaenoic acid (DPA) (0.48 ± 0.10 at T0 vs. 0.65 ± 0.16 at T1) and EPA (0.32 ± 0.09 at T0 vs. 0.46 ± 0.10 at T1). At the same time, LA and AA significantly increased, whereas oleic acid was drastically decreased (*p* < 0.0001 for all trends). Total ω6 and ω3 FAs significantly increased after intervention compared to baseline (*p* < 0.0001).

**Table 2:** Whole blood fatty acid profile in children (*n* = 15) with MIS-C supplemented with DHA (Group 1).

FA	Common name	% w/w(SD) at T0 in Group 1	% w/w(SD) at T1 in Group 1	% w/w(SD) at T2 in Group 1	*p* value
16:0	Palmitic acid	27.92 (1.66)^a^	26.53 (2.08)^b^	25.29 (1.70)^b^	0.0004
18:0	Stearic acid	10.15 (1.09)^a^	14.07 (1.16)^b^	12.46 (1.34)^c^	<0.0001
20:0	Arachidic acid	0.41 (0.10)^a^	0.57 (0.08)^b^	0.44 (0.11)^a^	0.0001
22:0	Behenic acid	1.04 (0.22)^a^	1.86 (0.30)^b^	1.59 (0.24)^c^	<0.0001
24:0	Lignoceric acid	1.72 (0.55)^a^	3.21 (0.70)^b^	2.64 (0.59)^b^	<0.0001
16:1	Palmitoleic acid	3.14 (0.83)^a^	1.28 (0.37)^b^	1.20 (0.54)^b^	<0.0001
18:1 n-9	Oleic acid	27.07 (3.39)^a^	16.13 (2.15)^b^	18.58 (3.20)^b^	<0.0001
18:1 n-7	7-Octadecenoic acid	1.65 (0.28)^a^	1.66 (0.37)^a,b^	1.39 (0.32)^b^	0.048
20:1	Eicosenoic acid	0.20 (0.13)	0.16 (0.04)	0.18 (0.04)	0.28
22:1	Eruric acid	0.08 (0.03)^a,b^	0.13 (0.08)^a^	0.04 (0.06)^b^	0.015
24:1	Nervonic acid	2.11 (0.58)^a^	2.97 (0.60)^b^	2.56 (0.47)^b^	0.0003
20:3 n-9	Eicosatrienoic acid	0.17 (0.10)	0.14 (0.04)	0.13 (0.03)	0.67
18:2 n-6	Linoleic acid (LA)	12.57 (2.22)^a^	14.56 (1.58)^b^	17.71 (2.82)^c^	<0.0001
18:3 n-6	Gamma linolenic acid	0.68 (0.41)^a^	0.25 (0.18)^a,b^	0.19 (0.08)^b^	0.001
20:3 n-6	Dihomogammalinolenic acid	1.13 (0.30)^a^	1.43 (0.38)^a,b^	1.34 (0.20)^b^	0.018
20:4 n-6	Arachidonic acid (AA)	6.60 (1.21)^a^	9.05 (1.79)^b^	9.29 (2.20)^b^	<0.0001
22:4 n-6	Adrenic acid	0.84 (0.26)^a^	1.13 (0.42)^b^	1.06 (0.32)^a,b^	0.017
22:5 n-6	Docosapentaenoic acid (DPA) n-6	0.36 (0.08)^a^	0.79 (0.32)^b^	0.33 (0.09)^a^	<0.0001
18:3 n-3	Alpha linolenic acid (ALA)	0.20 (0.09)	0.29 (0.16)^a^	0.21 (0.08)	0.074
20:5 n-3	Eicosapentaenoic acid (EPA)	0.32 (0.09)^a^	0.46 (0.10)^b^	0.41 (0.08)^b^	0.0002
22:5 n-3	Docosapentaenoic acid (DPA)	0.48 (0.10)^a^	0.65 (0.16)^b^	0.60 (0.20)^a.b^	0.037
22:6 n-3	Docosahexaenoic acid (DHA)	1.19 (0.25)^a^	2.67 (0.78)^b^	2.37 (0.87)^b^	<0.0001
SAT		41.23 (2.42)^a^	46.23 (3.13)^b^	42.42 (2.36)^a^	<0.0001
MONO		34.24 (3.61)^a^	22.33 (2.05)^b^	23.94 (3.38)^b^	<0.0001
POLY		24.52 (2.75)^a^	31.43 (3.09)^b^	33.64 (2.33)^b^	<0.0001
U.I.		108.56 (6.99)^a^	124.07 (10.84)^b^	127.31 (9.08)^b^	<0.0001
n-6		22.17 (2.71)^a^	27.21 (2.76)^b^	29.93 (2.10)^c^	<0.0001
n-3		2.18 (0.35)^a^	4.08 (0.80)^b^	3.59 (1.13)^b^	<0.0001
n-6/n-3		10.42 (2.05)^a^	6.88 (1.31)^b^	9.12 (2.79)^a^	<0.0001
DHA/AA		0.18 (0.3)^a^	0.31 (0.10)^b^	0.25 (0.06)^c^	<0.0001
EPA/AA		0.05 (0.02)	0.05 (0.01)	0.05 (0.01)	0.08
O3I		2.30 (0.30)^a^	4.10 (0.88)^b^	3.70 (1.02)^b^	<0.0001

Data are expressed as mean (standard deviation [SD]) of FA of the relative percentage (weight/weight) of all FA considered, analyzed as described in Methods. Statistical analysis performed: ANOVA for repeated measures with Bonferroni Correction and Friedman test with Dunn’s post-hoc test with multiple comparisons, for normally and not normally distributed variables, respectively. Values who do not share the same suffix (abc) are significantly different for *p*-value < 0.05. SAT, saturated fatty acids; MONO, monounsaturated fatty acids; POLY, polyunsaturated fatty acids; UI, Unsaturation Index; O3I, Omega-3 Index.

At T2 in Group 1, we observed only a slight reduction of EPA, DHA and DPA respect to T1, without significance. At 6 months, mean EPA and DHA values were 0.41 ± 0.08 and 2.37 ± 0.87, respectively, which remain significantly higher compared to baseline (*p* = 0.0002 and *p* < 0.0001, respectively). Regarding ω6 FA the significant trend toward an increase of mean levels persisted at T2 (22.17 ± 2.71, 27.21 ± 2.76 and 29.93 ± 2.10 at T0, T1 and T2, respectively, *p* < 0.0001). In fact, at T2 LA significantly increases compared to both T1 and T0, while AA was significantly higher compared to baseline. Oleic acid values at T2 significantly rose compared to T1, although they were still significantly lower than baseline. Overall, the observed changes are also reflected in the ω6/ω3 ratio, which significantly decreased from 10.42 ± 2.05 to 6.88 ± 1.31 at T1 and then significantly rose to 9.12 ± 2.79 at T2 (*p* < 0.0001), although remaining lower than baseline mean value.

Table 3 shows changes over time of FA profile in Group 2. As per Group 1, Group 2 experienced a significant increase of ω6 FA (20.26 ± 4.12 at T0, 27.91 ± 3.74 at T1, and 30.48 ± 2.19 at T2, *p* < 0.0001), with LA and AA being significant higher at T1 and T2 compared to baseline (*p* < 0.0001). There was a dramatic reduction of oleic acid from T0 to T1, and the trend persisted at T2 although without significance. Regarding ω3 FA, only ALA significantly increased from 0.17 ± 0.06 at baseline to 0.36 ± 0.16 at T1 (*p* = 0.0003), while DHA experienced a significant enrichment only at T2 compared to baseline (1.70 ± 0.43 at T2 vs1.09 ± 0.51 at T0, respectively). Interestingly, ω6/ω3 and DHA/AA ratios as well as O3I, did not significantly change over time in Group 2.

**Table 3:** Whole blood fatty acid profile in children (*n* = 15) with MIS-C with NO DHA supplementation (Group 2).

FA	Common name	% w/w(SD) at T0 in Group 2	% w/w(SD) at T1 in Group 2	% w/w(SD) at T2 in Group 2	*p* value
16:0	Palmitic acid	29.43 (2.45)^a^	24.56 (2.12)^b^	24.39 (0.9)^b^	0.0002
18:0	Stearic acid	10.62 (1.57)^a^	12.74 (1.34)^b^	11.84 (0.65)^b^	<0.0001
20:0	Arachidic acid	0.39 (0.12)^a^	0.54 (0.09)^b^	0.46 (0.05)^a,b^	0.0023
22:0	Behenic acid	1.06 (0.21)^a^	1.62 (0.20)^b^	1.52 (0.19)^b^	<0.0001
24:0	Lignoceric acid	1.67 (0.44)^a^	2.74 (0.47)^b^	2.66 (0.37)^b^	<0.0001
16:1	Palmitoleic acid	2.77 (1.21)^a^	1.09 (0.25)^b^	1.26 (0.42)^b^	<0.0001
18:1 n-9	Oleic acid	27.67 (4.27)^a^	21.64 (3.97)^b^	20.15 (1.81)^b^	<0.0001
18:1 n-7	7-Octadecenoic acid	1.63 (0.32)^a^	1.44 (0.24)^a^	1.31 (0.21)^b^	0.0023
20:1	Eicosenoic acid	0.19 (0.08)	0.18 (0.08)	0.16 (0.04)	0.22
22:1	Eruric acid	0.07 (0.05)	0.11 (0.15)	0.11 (0.15)	0.14
24:1	Nervonic acid	2.00 (0.44)^a^	2.75 (0.45)^b^	2.74 (0.33)^b^	<0.0001
20:3 n-9	Eicosatrienoic acid	0.15 (0.09)	0.12 (0.04)	0.16 (0.06)	0.14
18:2 n-6	Linoleic acid (LA)	12.70 (2.27)^a^	17.92 (2.66)^b^	19.29 (1.94)^b^	<0.0001
18:3 n-6	Gamma-linolenic acid	0.33 (0.31)	0.29 (0.14)	0.24 (0.09)	0.074
20:3 n-6	Dihomogammalinolenic acid	0.94 (0.32)^a^	1.09 (0.18)^a^	1.30 (0.24)^b^	0.0034
20:4 n-6	Arachidonic acid (AA)	5.31 (1.84)^a^	7.35 (1.38)^b^	8.30 (1.55)^b^	0.0001
22:4 n-6	Adrenic acid	0.65 (0.29)^a^	0.91 (0.26)^b^	1.04 (0.26)^b^	0.0003
22:5 n-6	Docosapentaenoic acid (DPA) n-6	0.33 (0.16)	0.34 (0.16)	0.31 (0.08)	0.98
18:3 n-3	Alfa-linolenic acid (ALA)	0.17 (0.06)^a^	0.36 (0.16)^b^	0.20 (0.06)^a^	0.0003
20:5 n-3	Eicosapentaenoic acid (EPA)	0.41 (0.11)^a^	0.28 (0.10)^b^	0.33 (0.09)^a,b^	0.0088
22:5 n-3	Docosapentaenoic acid (DPA)	0.41 (0.22)	0.48 (0.10)	0.57 (0.13)	0.082
22:6 n-3	Docosahexaenoic acid (DHA)	1.09 (0.51)^a^	1.44 (0.47)^a,b^	1.70 (0.43)^b^	0.003
SAT		43.17 (3.34)^a^	42.20 (3.22)^a,b^	40.88 (1.46)^b^	0.038
MONO		34.35 (4.64)^a^	27.20 (3.71)^b^	25.68 (2.16)^b^	<0.0001
POLY		22.48 (4.60)^a^	30.60 (4.03)^b^	33.44 (2.24)^c^	<0.0001
U.I.		100.61 (12.45)^a^	115.86 (10.40)^b^	123.57 (6.84)^b^	<0.0001
n-6		20.26 (4.12)^a^	27.91 (3.74)^b^	30.48 (2.19)^c^	<0.0001
n-3		2.07 (0.75)^a^	2.57 (0.57)^a,b^	2.80 (0.56)^b^	0.022
n-6/n-3		10.42 (2.72)	11.24 (2.11)	11.27 (2.27)	0.34
DHA/AA		0.20 (0.05)	0.19 (0.05)	0.21 (0.04)	0.49
EPA/AA		0.09 (0.05)^a^	0.04 (0.01)^b^	0.04 (0.01)^b^	<0.0001
O3I		2.30 (0.59)	2.54 (0.58)	2.88 (0.53)	0.074

Data are expressed as mean (standard deviation [SD]) of FA of the relative percentage (weight/weight) of all FA considered, analyzed as described in Methods. Statistical analysis performed: ANOVA for repeated measures with Bonferroni Correction and Friedman test with Dunn’s post-hoc test with multiple comparisons, for normally and not normally distributed variables, respectively. Values that do not share the same suffix (abc) are significantly different (*p*-value < 0.05). SAT, saturated fatty acids; MONO, monounsaturated fatty acids; POLY, polyunsaturated fatty acids; UI, Unsaturation Index; O3I, Omega-3 Index.

Table 4 shows the significant changes of FAs between groups, expressed as *Δ* values. After intervention at T1, there was a significantly greater increase of LA in Group 2 compared to Group 1 (*p* = 0.01), however, this difference was no longer statistically significant after adjustment (*p*_adj_ = 0.19). On the contrary the ΔDHA at T1 was significantly higher in Group 1 respect to Group 2 (unadjusted *p* = 0.0001), and this difference remained statistically significant after adjustment (*p*_adj_ = 0.015). Interestingly, the EPA trend at T1 is significantly different among groups as it increases in Group 1 while it decreases in Group 2 [0.15(0.1) vs. − 0.13(0.1), *p* < 0.0001]. This difference remained statistically significant after adjustment (*p*_adj_ = 0.0024). Overall, the ΔT1-T0 increase in total n-3 was significantly higher in Group 1 than Group 2 [1.90(0.9) vs. 0.49(0.8), *p* < 0.0001 and *p*_adj_ = 0.005].

**Table 4:** Significant changes (expressed as ΔT1-T0, ΔT2-T0, and ΔT2-T1) for each fatty acid between Group 1 (suppl DHA) vs. Group 2 (no DHA).

FA	ΔT1-T0 Group 1	ΔT1-T0 Group 2	*p* value	*p*_adj_ value	ΔT2-T0 Group 1	ΔT2-T0 Group 2	*p* value	*p*_adj_ value	ΔT2-T1 Group 1	ΔT2-T1 Group 2	*p* value	*p*_adj_ value
	% w/w(SD)	% w/w(SD)			% w/w(SD)	% w/w(SD)			% w/w(SD)	% w/w(SD)		
16:0	−1.39 (1.7)	−4.87 (3.8)	**0.003**	0.089	−2.63 (2.2)	−5.04 (2.9)	**0.02** ^**§**^	0.32	−1.24 (2.3)	−0.17 (2.2)	0.20	0.24
18:0	3.92 (1.6)	2.11 (1.6)	**0.004**	0.23	2.31 (1.7)	1.22 (1.3)	**0.01** ^**§**^	0.69	−1.61 (1.2)	−0.89 (1.3)	0.14	0.33
20:0	0.16 (0.2)	0.15 (0.1)	0.51^§^	0.41	0.03 (0.2)	0.07 (0.1)	0.83^**§**^	0.47	−0.13 (0.1)	−0.08 (0.1)	0.13^§^	0.98
22:0	0.82 (0.4)	0.56 (0.3)	**0.05**	0.43	0.55 (0.3)	0.46 (0.3)	0.44	0.67	−0.27 (0.3)	−0.10 (0.2)	0.11	0.62
24:0	1.49 (0.8)	1.07 (0.5)	0.10^§^	0.45	0.92 (0.8)	1.00 (0.5)	0.74	0.83	−0.57 (0.8)	−0.08 (0.5)	**0.03** ^**§**^	0.32
16:1	−1.86 (0.9)	−1.69 (1.2)	0.37^§^	0.71	−1.94 (1.0)	−1.51 (1.0)	0.24	0.39	−0.09 (0.5)	0.18 (0.4)	0.13	0.31
18:1 n-9	−10.94 (4.3)	−6.04 (5.3)	**0.01**	0.17	−8.49 (4.5)	−7.52 (4.0)	0.25^**§**^	0.92	2.44 (3.6)	−1.49 (3.6)	**0.006**	0.09
18:1 n-7	0.02 (0.5)	−0.19 (0.3)	0.18	0.82	−0.26 (0.4)	−0.31 (0.3)	0.67	0.12	−0.27 (0.5)	−0.13 (0.2)	0.26	0.18
20:1	−0.04 (0.1)	−0.02 (0.1)	0.99^§^	0.64	−0.02 (0.1)	−0.03 (0.1)	0.06^**§**^	0.93	0.02 (0.1)	−0.02 (0.1)	0.24	0.57
22:1	0.05 (0.1)	0.03 (0.1)	0.37^§^	0.66	−0.04 (0.1)	−0.03 (0.1)	0.58	0.58	−0.09 (0.1)	−0.06 (0.2)	0.37^§^	0.99
24:1	0.86 (0.8)	0.75 (0.6)	0.65	0.93	0.45 (0.7)	0.74 (0.5)	0.30^**§**^	0.24	−0.40 (0.6)	0.01 (0.6)	0.08	0.27
20:3 n-9	−0.03 (0.1)	−0.03 (0.1)	0.80^§^	0.67	−0.04 (0.1)	0.01 (0.1)	0.07^**§**^	0.55	−0.02 (0.1)	0.04 (0.1)	**0.019**	0.85
18:2 n-6	1.99 (2.8)	5.23 (3.6)	**0.01**	0.19	5.14 (3.5)	6.59 (2.5)	0.20	0.56	3.15 (3.5)	1.36 (2.2)	0.10	0.37
18:3 n-6	−0.42 (0.4)	−0.04 (0.3)	**0.02** ^**§**^	**0.0048**	−0.49 (0.4)	−0.10 (0.3)	**0.005** ^**§**^	**0.0037**	−0.07 (0.2)	−0.06 (0.1)	0.84	0.62
20:3 n-6	0.30 (0.5)	0.15 (0.4)	0.31	0.31	0.22 (0.3)	0.36 (0.4)	0.26	0.94	−0.09 (0.3)	0.21 (0.2)	**0.0066**	0.16
20:4 n-6	2.46 (1.9)	2.05 (2.4)	0.60	0.55	2.69 (1.9)	3.00 (2.4)	0.69	0.84	0.23 (2.4)	0.95 (1.5)	0.34	0.68
22:4 n-6	0.29 (0.4)	0.26 (0.3)	0.85	0.52	0.22 (0.3)	0.39 (0.3)	0.11^**§**^	0.77	−0.06 (0.3)	0.13 (0.2)	0.08	0.26
22:5 n-6	0.42 (0.3)	0.01 (0.2)	**<0.0001** ^**§**^	**0.016**	−0.03 (0.1)	−0.02 (0.2)	0.28^**§**^	0.66	−0.45 (0.3)	−0.03 (0.2)	**<0.0001** ^**§**^	**0.0072**
18:3 n-3	0.10 (0.2)	0.20 (0.2)	0.089^§^	0.74	0.01 (0.1)	0.04 (0.1)	0.53	0.44	−0.08 (0.2)	−0.16 (0.2)	**0.029** ^**§**^	0.89
20:5 n-3	0.15 (0.1)	−0.13 (0.1)	**<0.0001**	**0.0024**	0.09 (0.1)	−0.08 (0.1)	**0.0005**	**0.0028**	−0.05 (0.1)	0.05 (0.1)	**0.020**	0.65
22:5 n-3	0.17 (0.2)	0.07 (0.2)	0.22	0.12	0.12 (0.2)	0.16 (0.2)	0.54^**§**^	0.74	−0.05 (0.3)	0.09 (0.2)	0.13	0.30
22:6 n-3	1.49 (0.8)	0.35 (0.6)	**0.0001**	**0.015**	1.18 (0.8)	0.61 (0.7)	**0.04**	0.24	−0.31 (0.8)	0.26 (0.5)	**0.032**	0.18
SAT	5.00 (3.4)	−0.97 (4.8)	**0.0005**	0.07	1.18 (3.6)	−2.30 (3.8)	**0.008** ^**§**^	0.41	−3.82 (3.7)	−1.32 (3.6)	**0.030** ^**§**^	0.20
MONO	−11.91 (4.2)	−7.15 (5.1)	**0.009**	0.13	−10.30 (4.8)	−8.67 (4.2)	0.32	0.73	1.61 (3.4)	−1.52 (3.1)	**0.014**	0.09
POLY	6.91 (3.5)	8.12 (6.6)	0.53	0.91	9.12 (3.1)	10.96 (5.3)	0.25	0.74	2.21 (3.9)	2.84 (3.7)	0.65	0.81
U.I.	15.51 (11.2)	15.25 (17.3)	0.96	0.72	18.74 (9.4)	22.96 (15.7)	0.38	0.98	3.24 (13.0)	7.71 (11.1)	0.32	0.64
n-6	5.04 (2.9)	7.66 (6.1)	0.15	0.54	7.75 (2.9)	10.22 (4.6)	0.09	0.49	2.71 (3.3)	2.57 (3.3)	0.90	0.91
n-3	1.90 (0.9)	0.49 (0.8)	**<0.0001**	**0.005**	1.41 (1.0)	0.73 (0.9)	0.06	0.21	−0.49 (1.3)	0.23 (0.7)	0.06	0.22
n-6/n-3	−3.54 (2.0)	0.82 (2.6)	**<0.0001**	**0.0004**	−1.30 (2.1)	0.86 (2.6)	**0.02**	0.12	2.25 (2.6)	0.03 (2.4)	**0.024**	**0.04**
DHA/AA	0.12 (0.1)	−0.01 (0.05)	**<0.0001**	**0.0098**	0.07 (0.1)	0.002 (0.1)	**0.0017**	0.12	−0.05 (0.1)	0.01 (0.05)	**0.0012** ^**§**^	**0.023**
EPA/AA	0.003 (0.02)	−0.05 (0.1)	**<0.0001** ^**§**^	0.092	0.004 (0.0)	−0.05 (0.0)	**0.0003** ^**§**^	0.07	−0.01 (0.01)	0.002 (0.02)	0.051	0.72
O3I	1.80 (0.9)	0.24 (0.9)	**<0.0001**	**0.0077**	1.40 (0.9)	0.59 (0.9)	**0.019**	0.11	−039 (1.0)	0.34 (0.8)	**0.040**	0.22

Data are expressed as mean ± standard deviation (SD) of FA of the relative percentage (weight/weight) of all FA considered. *p* value calculated with unpaired t test for normally distributed variables; ^§^*p* value calculated with Mann Whitney U test for not normally distributed variables. p_adj_ *p*-value calculated adjusting for Propensity Score of baseline data. *p*-values in bold indicate statistical significance (*p* < 0.05). SAT, saturated fatty acids; MONO, monounsaturated fatty acids; POLY, polyunsaturated fatty acids; UI, Unsaturation Index; O3I, Omega-3 Index.

In Group 1 the ΔT2-T0 for EPA remained positive and was significantly different compared to Group 2 [0.09(0.1) vs. −0.08(0.1) unadjusted *p* = 0.0005], and this difference remained statistically significant after adjustment (*p*_adj_ = 0.0028). Conversely, the ΔT2–T0 for DHA was significantly greater in Group 1 compared to Group 2 in the unadjusted analysis [1.18(0.8) vs. 0.61(0.7); *p* = 0.04], but this difference was no longer statistically significant after adjustment (adjusted *p* = 0.24). A significantly different pattern of n-6/n-3 ratio was observed between the two groups at each timepoint.

**Figure 2:**
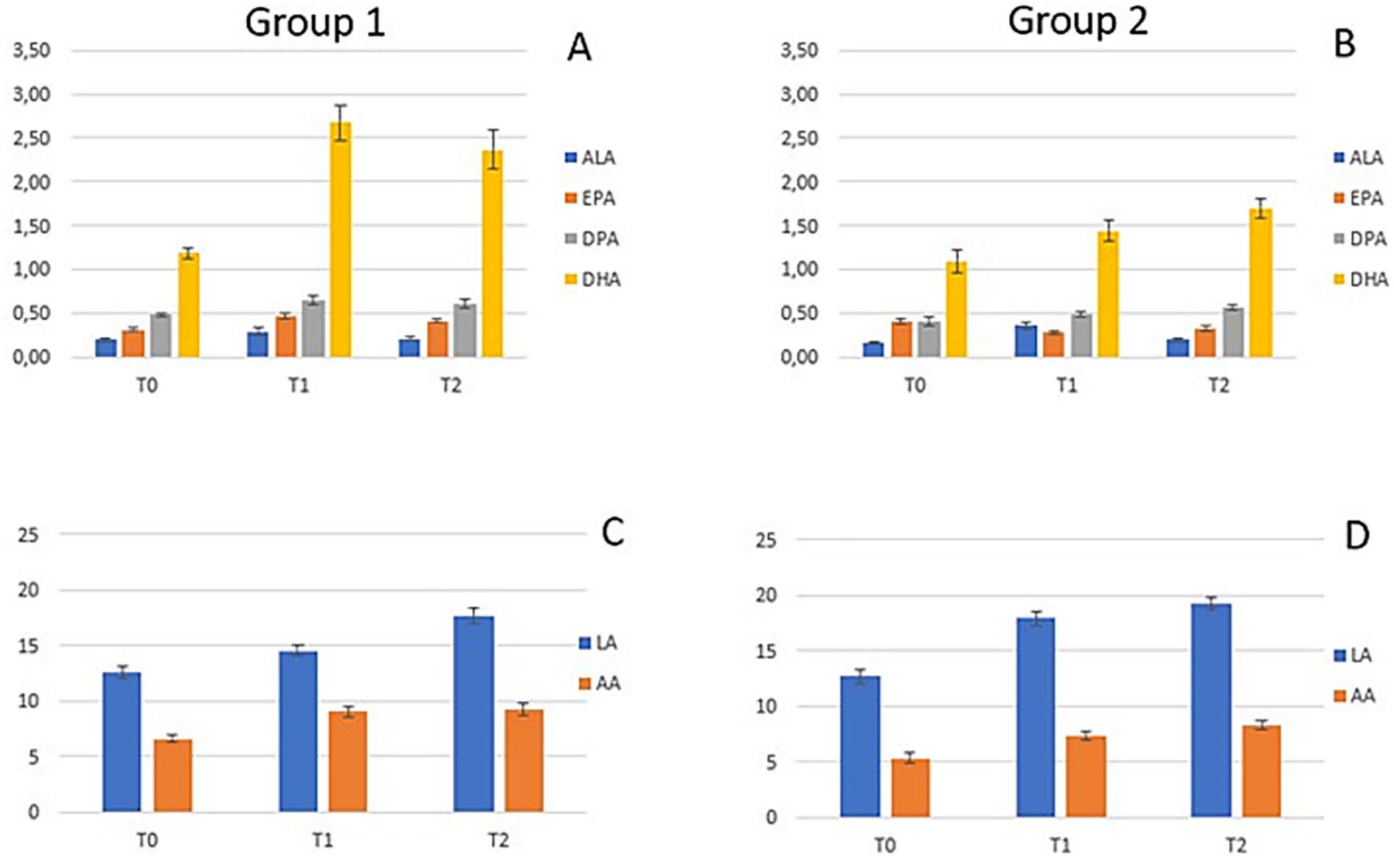
Fatty acids levels at time points T0, T1, and T2 in Group 1 **(A, C)** and Group 2 **(B, D)**. **(A)** Concentrations of n-3 (ALA, EPA, DPA, and DHA) in Group 1; **(B)** Concentrations of n-3 (ALA, EPA, DPA, and DHA) in Group 2; **(C)** Concentrations of n-6 (LA and AA) in Group 1; **(D)** Concentrations of n-6 (LA and AA) in Group 2.

Figure 2 shows the levels of ω3 and ω6 FA measured at the different time points in the two groups. Both groups showed an increase in ω3 but in Group 1 the increase was more marked. Regarding DHA levels, at T1 Group 1 showed a +*Δ*124% increase vs. +Δ32% in Group 2, and at T2 + Δ 99% vs. +Δ56% increase compared to baseline, respectively (Figures 2A,B). Specifically, DHA in Group 1 (Figure 2A) showed a typical bell-shaped trend due to supplementation followed by washout, while Group 2 exhibits a constant upward trend but never matches DHA levels of Group 1.

FAs of the ω6 series showed a similar trend for both groups (Figures 2C,D), specifically both LA and AA increased from T0 to T2.

The FAs values at the three time points (T0, T1 and T2) in the two groups were compared with reference values found in the literature in pediatric groups without current acute inflammation (21–25) (Supplementary Table 2). We observed that LA and AA across all timepoints in both groups were lower or borderline low compared to reference values. Regarding EPA mean values in Group 1 at T1 and T2 were higher compared to reference values, apart from Crippa et al., while in Group 2 values were borderline high. DHA mean values at baseline in both groups were depleted and lower compared to reference values, except from Ryan et al. In Group 1 at T1 and T2 there was a restoring to values similar to references, while this was not observed in Group 2 whose values remained lower than references, excluding value from Ryan et al. At T2 also ALA levels were higher in both groups compared to reference values.

Table 5 summarizes the biochemical parameters measured at T0 and T2. At T0, all parameters, except for albumin, were higher than reference values due to the inflammatory status. At T2, all parameters returned to the normal range.

**Table 5:** Whole blood values in Group 1 (*n* = 15) vs. Group 2 (*n* = 15) at T0 and T2.

Biochemistry	Group 1 at T0	Group 1 at T2	Group 2 at T0	Group 2 at T2	Reference values	*p* value^*^	Δ between groups (G2-G1) (95% CI)	Effect size (95% CI)
CRP (mg/L)	199.8 (105.6)	0.8 (1.1)	175.5 (88.3)	0.9 (0.5)	≤10 mg/L (55)	0.5	24.20 (−48.43, 96.83)	0.25
Ferritin (μg/L)^§^	1537.1 (1,881)	30.7 (12.7)	593.7 (504.2)	25.5 (14.4)	<30 μg/L [0–5 yo] <70 μg/L [5–18 yo] subjects with infection or inflammation (56)	0.14	290 (−53, 1,340)	0.27
ESR (mm/h)	51 (27.2)	5.6 (5.4)	59.9 (32.6)	9.1 (6.4)	≤20 mm/h (57)	0.68	−4.81 (−28.6, 19.3)	−0.16
IL-6 (ng/L) §	31.88 (65.6)	0.37 (0.01)	17.94 (17.2)	0.37 (0.01)	7 (ng/L) (58)	0.63	1.25 (−8.9, 31.6)	0.07
Albumin (g/dL) §	2.6 (0.4)	4.5 (0.3)	2.8 (0.5)	7.1 (10.5)	35–50 g/L (59)	0.11	−0.40 (−0.8, 0.1)	0.29
D-Dimer (μg/L) §	3763.5 (3535.7)	439.8 (294.9)	5736.7 (6062.4)	425.5 (402.6)	<500 μg/L (60)	0.5	−758 (−3,366, 1,010)	0.12
Fibrinogen (g/L) §	6.5 (0.8)	4.1 (0.7)	5.6 (1.5)	3.9 (0.6)	2–4 g/L (61)	0.12	−0.46 (−1.26, 0.30)	0.28
Glucose (mg/dL)	111.53 (37.11)	87.47 (7.93)	118.36 (30.81)	86.53 (5.13)	<100 mg/dL (62)	0.57	−7.22 (−33.63, 19.19)	−0.21
Cholesterol total (mg/dL)	129.27 (46.9)	152.60 (29.5)	145.1 (43.1)	151.2 (24.9)	<170 mg/dL (62)	0.31	−17.76 (−53.6, 18.09)	−0.38
HDL (mg/dL)	15.13 (10.6)	48.53 (10.3)	23.1 (15.1)	49.8 (7.1)	>45 mg/dL (62)	0.25	−6.97 (−19.30, 5.35)	−0.43
LDL (mg/dL)	70.25 (31.8)	86.84 (26.4)	86.9 (27.9)	86.4 (23.5)	<110 mg/dL (62)	0.37	−19.16 (−48.17, 9.84)	−0.56
Triglycerides (mg/dL)	227 (96.42)	89.93 (51.68)	207.29 (103.47)	74.67 (24.09)	<75 mg/dL [0–9 yo] <90 mg/dL [10–19 yo] (62)	0.82	9.21 (−73.47, 91.90)	0.09
TyG Index	9.30 (0.52)	8.16 (0.51)	9.28 (0.47)	8.03 (0.32)	<7.88 (63, 64)	0.72	−0.082 (−0.55, 0.38)	−0.14

Data are expressed as mean ± standard deviation (SD) of blood values. § not-normally distributed variables. ^*^p value of the difference (T2-T0) between groups, calculated according to unpaired t test and Mann Whitney U test for normally and not normally distributed variables, respectively. Delta (Δ) represents the between-group difference: for normally distributed variables, it reflects the difference in means (95% CI, unpaired t-test); for non-normally distributed variables, it reflects the Hodges–Lehmann estimate of the median difference (95% CI, Mann–Whitney test). Effect sizes are reported as Cohen’s d or non-parametric r where appropriate. The triglyceride–glucose (TyG) index was calculated as [ln(fasting triglycerides (mg/dL) fasting plasma glucose (mg/dL)/2)]. CRP, C-Reactive Protein; ESR, Erythrocyte Sedimentation Rate; HDL, High-Density Lipoprotein Cholesterol; IL-6, Interleukin-6; LDL, Low-Density Lipoprotein Cholesterol; TyG Index, Triglyceride-Glucose Index.

**Figure 3:**
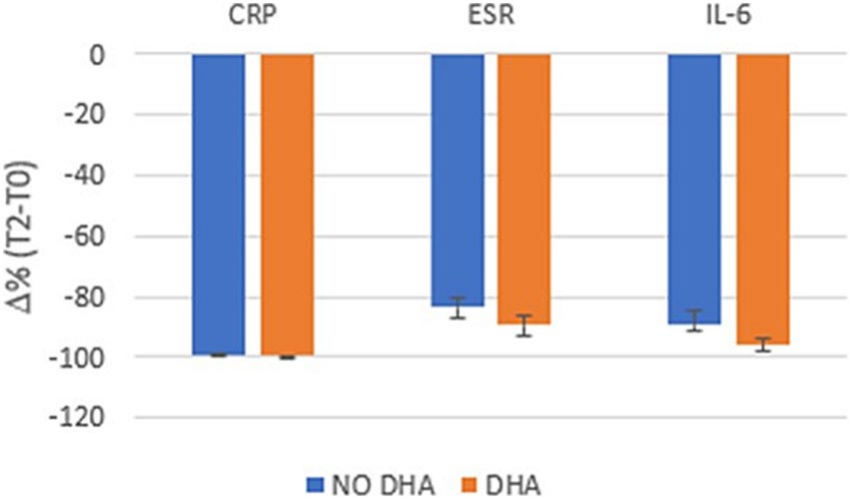
Delta (*Δ*)% reduction (T2-T0) of inflammatory markers in Group 1 (red color) and Group 2 (blue color).

With respect to the change in biochemical parameters, no significant differences were observed between the groups (Table 5). Figure 3 shows the reduction, expressed as *Δ* % (T2-T0), in the levels of the inflammatory markers measured in the two groups. CRP decreased similarly in Group 1 and 2, while ESR and IL-6 showed a greater change Δ % and a better tendency toward normalization in Group 1. Although the effect sizes reported in Table 5 are small (CRP: 0.25; ESR: −0.16; IL-6: 0.07), indicating that the observed differences between the two groups may have limited practical impact.

## 4 Discussion

Current evidence supports the use of ω3 FA supplementation in the prevention and treatment of a wide range of human diseases, including coronary artery disease, diabetes, hypertension, arthritis, and other inflammatory and autoimmune conditions (45, 46). In fact by triggering the production of SPMs, ω3 actively control the inflammatory process. These mediators reduce the severity of the inflammatory process and promote its active resolution by suppressing the overproduction of pro-inflammatory lipid-derived compounds and cytokines (47). In fact, inflammation is a process consisting of two phases: initiation and resolution. In the first phase, ω6 AA enters the cell through the phospholipid membrane and produces a series of molecules called eicosanoids which are involved in the inflammatory process (48). At the same time, the ω3 EPA, DHA and DPA also enter the cell, and act as substrates for specialized pro-resolving mediators (resolvins, protectins and maresins), modulating inflammation (26, 49). In adults, lower levels of DHA and O3I have been associated with a higher risk of adverse COVID-19 outcomes. In this study, we analyzed the FA profile and inflammation-related blood values in a cohort of children with MIS-C (29). At baseline during the acute phase, we confirmed low levels of ω6 fatty acids that could be attributed to the inflammatory process during which these fatty acids are converted to eicosanoids. After, both groups of children experienced a trend toward increase of total ω6 from baseline to 6 months. Regarding ω3 values, we observed depleted DHA stores at baseline in both groups, with mean values lower than reference values in pediatric age. This status might be explained by the body’s attempt to counterbalance ongoing inflammation.

Our objective was to evaluate whether supplementation with DHA restores normal fatty acid values and improve normalization of biochemical parameters. Our results show a significant increase in DHA and EPA blood values both at T1 and T2, after discontinuation of the supplementation, in the supplemented group compared to patients not supplemented. The DHA/AA ratio was significantly increased in Group 1 at T1 and persisted at T2, while no changes were observed in DHA/AA ratio in Group 2. Interestingly, the ω6/ω3 ratio was found to be higher in Group 2 compared to Group 1 during the follow up. At 3 months in Group 2, DHA and DPA mean values were not significantly changed compared to baseline. Only at T2 they experienced a slight restoration of DHA values, with mean values still significantly lower compared to both the supplemented group and values reported in the literature for healthy subjects. During follow-up, both groups showed a substantial decline in monounsaturated fatty acids, with oleic acid decreasing most prominently. Our findings of altered fatty acid profiles in MIS-C patients are consistent with previous observations in COVID-19, where SARS-CoV-2 infection induced perturbations in fatty acid biosynthesis pathways (18). The improvement in DHA levels may help counteract sphingolipid disturbances and inflammatory lipid mediator imbalances (12, 13).

By looking at the biochemicals parameters, both groups showed complete resolution of inflammation at T2, which can be attributed to the physiological process that naturally follows the acute phase of the disease. Interestingly, while CRP decreased similarly in both groups, ESR and IL-6 showed a greater change and a better tendency toward normalization in the ω3 FA supplemented group. The anti-inflammatory effects of DHA supplementation may be particularly relevant in post-COVID inflammatory conditions. DHA-derived specialized pro-resolving mediators could help modulate platelet-activating factor and endocannabinoid pathways (12), while counteracting oxidative stress and matrix metalloproteinase activation (19).

Given its exploratory nature, this study was designed as a pilot investigation to gather initial data on the role of DHA supplementation in systemic inflammatory conditions in pediatric age groups and guide future research. Studies in adults with severe COVID-19 showed the benefits of early use of ω3 PUFA supplementation (50–52). While nutritional interventions involving PUFAs have been studied in pediatric inflammatory conditions such as IBD (53), evidence is currently lacking in Kawasaki disease and in critically ill children. The current body of evidence is largely derived from adult studies, highlighting the need for pediatric-focused research of an early use of ω3 PUFA in the hospital setting and during follow-up in critically ill pediatric patients. One of the main limitations of this work is therefore the pilot study design. As such, the sample size was relatively small due to high drop-out rate, and the study was not powered to detect small or moderate effects with high statistical certainty. While the results are promising and provide valuable preliminary insights, they should be interpreted with caution. Also, the lack of blinding was a consequence of the emergency conditions during the COVID-19 pandemic, which required a rapid and pragmatic approach to recruitment and intervention delivery. While necessary in this context, this limitation should be considered when interpreting the findings.

Another limitation of our study was the impossibility to directly measure SPMs: while it is commonplace in the literature to report plasmatic concentrations of these bioactive metabolites, it is unlikely that their blood concentrations may reflect the actual concentrations at the inflammatory site(s), given that they undergo rapid local and systemic (possibly hepatic) biotransformation (54). Consequently, interpretations regarding SPM activity remain speculative. Moreover, circulating fatty acid levels, although useful, represent only surrogate markers and may not fully capture the complexity or activity of SPM-mediated resolution pathways. Finally, the interpretation of the results may be complicated by the fact that values of AA or DHA (and LA or EPA) at T0 are in fact the result of two separate factors: first the (higher or lower) values present before the acute phase of the disease, and second the extent of decrease resulting from the more or less significant use of these substrate for the biosynthesis of pro-inflammatory (in the case of AA and LA) or anti-inflammatory (for DHA and possibly EPA) biologically active metabolites, whose balance may ultimately affect the course of the inflammatory response. Future directions could include the implementation of targeted assays for SPMs to better characterize their dynamics and contributions to inflammatory resolution.

In conclusion, ω3 PUFA play a pivotal role in the blood fatty acid profile of children with MIS-C. The high inflammatory response associated with this condition leads to an increased endogenous utilization of these fatty acids, as evidenced by their marked decrease in circulating levels. Although the SARS-CoV-2 pandemic has ended and MIS-C cases have dramatically declined, this syndrome represents a valuable model of acute inflammatory multisystem disease. Monitoring fatty acid status in these pediatric patients may provide clinically meaningful insights into systemic inflammation both after the acute phase and during follow-up.

Our findings suggest that DHA supplementation may be associated with the resolution of inflammatory status in children with acute inflammatory multisystem disease during the stable and recovery phase. The observed improvement in fatty acid status, along with a trend toward normalization of biochemical parameters, might contribute to a more favorable recovery trajectory, potentially enhancing resilience to subsequent inflammatory challenges. These results underscore the potential role of n-3 PUFAs in pediatric acute inflammatory syndromes. Further adequately powered, controlled studies are needed to confirm these observations and to evaluate the potential role of early n-3 PUFA supplementation during the stable and recovery phases in critically ill pediatric patients.

## Data Availability

The datasets presented in this article are not readily available because data sharing must be carefully authorized per data privacy policy. Requests to access the datasets should be directed to GF, giulia.fiore@unimi.it.

## References

[1] Feldstein LR Rose EB Horwitz SM Collins JP Newhams MM Son MBF. Multisystem inflammatory syndrome in U.S. children and adolescents. N Engl J Med. (2020) 383:334–46. doi: 10.1056/NEJMoa2021680, PMID: 32598831 PMC7346765

[2] CDC. Multisystem inflammatory syndrome (MIS). Centers for Disease Control and Prevention (2020) Available online at: https://www.cdc.gov/mis/mis-c/hcp_cstecdc/index.html (Accessed January 15, 2024).

[3] Payne AB Gilani Z Godfred-Cato S Belay ED Feldstein LR Patel MM. Incidence of multisystem inflammatory syndrome in children among US persons infected with SARS-CoV-2. JAMA Netw Open. (2021) 4:e2116420. doi: 10.1001/jamanetworkopen.2021.16420, PMID: 34110391 PMC8193431

[4] Holm M Hartling UB Schmidt LS Glenthøj JP Kruse A Rytter MH. Multisystem inflammatory syndrome in children occurred in one of four thousand children with severe acute respiratory syndrome coronavirus 2. Acta Paediatr. (2021) 110:2581–3. doi: 10.1111/apa.15985, PMID: 34129731 PMC8444758

[5] Lee PY Day-Lewis M Henderson LA Friedman KG Lo J Roberts JE. Distinct clinical and immunological features of SARS-CoV-2-induced multisystem inflammatory syndrome in children. J Clin Invest. (2020) 130:5942–50. doi: 10.1172/JCI141113, PMID: 32701511 PMC7598077

[6] Beckmann ND Comella PH Cheng E Lepow L Beckmann AG Tyler SR. Downregulation of exhausted cytotoxic T cells in gene expression networks of multisystem inflammatory syndrome in children. Nat Commun. (2021) 12:4854. doi: 10.1038/s41467-021-24981-1, PMID: 34381049 PMC8357784

[7] Whittaker E Bamford A Kenny J Kaforou M Jones CE Shah P. Clinical characteristics of 58 children with a pediatric inflammatory multisystem syndrome temporally associated with SARS-CoV-2. JAMA. (2020) 324:259–69. doi: 10.1001/jama.2020.10369, PMID: 32511692 PMC7281356

[8] Stasiak A Perdas E Smolewska E. Risk factors of a severe course of pediatric multi-system inflammatory syndrome temporally associated with COVID-19. Eur J Pediatr. (2022) 181:3733–8. doi: 10.1007/s00431-022-04584-8, PMID: 35948653 PMC9364844

[9] Jaxybayeva I Boranbayeva R Bulegenova M Urazalieva N. Clinical and immunological features in children with multisystem inflammatory syndrome associated with SARS-CoV-2. Acta Biomed. (2023) 94:e2023016. doi: 10.23750/abm.v94i2.13777, PMID: 37092638 PMC10210569

[10] Haslak F Gunalp A Kasapcopur O. A cursed goodbye kiss from severe acute respiratory syndrome-coronavirus-2 to its pediatric hosts: multisystem inflammatory syndrome in children. Curr Opin Rheumatol. (2023) 35:6–16. doi: 10.1097/BOR.0000000000000910, PMID: 36094472

[11] Rowley AH Shulman ST Arditi M. Immune pathogenesis of COVID-19-related multisystem inflammatory syndrome in children. J Clin Invest. (2020) 130:5619–21. doi: 10.1172/JCI143840, PMID: 32870815 PMC7598032

[12] de Carvalho JCS da Silva-Neto PV Toro DM Fuzo CA Nardini V Pimentel VE. The interplay among glucocorticoid therapy, platelet-activating factor and endocannabinoid release influences the inflammatory response to COVID-19. Viruses. (2023) 15:573. doi: 10.3390/v15020573, PMID: 36851787 PMC9959303

[13] Toro DM da Silva-Neto PV de Carvalho JCS Fuzo CA Pérez MM Pimentel VE. Plasma sphingomyelin disturbances: unveiling its dual role as a crucial Immunopathological factor and a severity prognostic biomarker in COVID-19. Cells. (2023) 12:1938. doi: 10.3390/cells12151938, PMID: 37566018 PMC10417089

[14] Fuzo CA Fraga-Silva TFC Maruyama SR Bastos VAF Rogerio LA Takamiya NT. The turning point of COVID-19 severity is associated with a unique circulating neutrophil gene signature. Immunology. (2023) 169:323–43. doi: 10.1111/imm.13631, PMID: 36740582

[15] Zuccotti G Calcaterra V Mannarino S D’Auria E Bova SM Fiori L. Six-month multidisciplinary follow-up in multisystem inflammatory syndrome in children: an Italian single-center experience. Front Pediatr. (2022) 10:1080654. doi: 10.3389/fped.2022.1080654 36776681 PMC9909209

[16] Calcaterra V Bosoni P Dilillo D Mannarino S Fiori L Fabiano V. Impaired glucose-insulin metabolism in multisystem inflammatory syndrome related to SARS-CoV-2 in children. Children. (2021) 8:384. doi: 10.3390/children8050384, PMID: 34067965 PMC8152288

[17] Di Profio E Leone A Vizzuso S Fiore G Pascuzzi MC Agostinelli M. Longitudinal anthropometry and body composition in children with SARS-CoV-2-associated multisystem inflammatory syndrome. J Pediatr Gastroenterol Nutr. (2023) 76:505–11. doi: 10.1097/MPG.0000000000003705, PMID: 36689921 PMC10012841

[18] Pérez MM Pimentel VE Fuzo CA da Silva-Neto PV Toro DM Fraga-Silva TFC. Acetylcholine, fatty acids, and lipid mediators are linked to COVID-19 severity. J Immunol. (2022) 209:250–61. doi: 10.4049/jimmunol.2200079, PMID: 35768148

[19] da Silva-Neto PV de Carvalho JCS Pimentel VE Pérez MM Toro DM Fraga-Silva TFC. sTREM-1 predicts disease severity and mortality in COVID-19 patients: involvement of peripheral blood leukocytes and MMP-8 activity. Viruses. (2021) 13:2521. doi: 10.3390/v13122521, PMID: 34960790 PMC8708887

[20] Verduci E Risé P Di Profio E Fiori L Vizzuso S Dilillo D. Blood fatty acids profile in MIS-C children. Meta. (2021) 11:721. doi: 10.3390/metabo11110721, PMID: 34822379 PMC8624489

[21] Bonafini S Giontella A Tagetti A Bresadola I Gaudino R Cavarzere P. Fatty acid profile and desaturase activities in 7-10-year-old children attending primary School in Verona South District: association between Palmitoleic acid, SCD-16, indices of adiposity, and blood pressure. Int J Mol Sci. (2020) 21:3899. doi: 10.3390/ijms21113899, PMID: 32486144 PMC7312303

[22] Risé P Tragni E Ghezzi S Agostoni C Marangoni F Poli A. Different patterns characterize omega 6 and omega 3 long chain polyunsaturated fatty acid levels in blood from Italian infants, children, adults and elderly. Prostaglandins Leukot Essent Fatty Acids. (2013) 89:215–20. doi: 10.1016/j.plefa.2013.06.009, PMID: 23910046

[23] Crippa A Agostoni C Mauri M Molteni M Nobile M. Polyunsaturated fatty acids are associated with behavior but not with cognition in children with and without ADHD: an Italian study. J Atten Disord. (2018) 22:971–83. doi: 10.1177/1087054716629215, PMID: 26861157

[24] van der Wurff ISM von Schacky C Bergeland T Leontjevas R Zeegers MP Jolles J. Effect of 1 year krill oil supplementation on cognitive achievement of Dutch adolescents: a double-blind randomized controlled trial. Nutrients. (2019) 11:1230. doi: 10.3390/nu11061230, PMID: 31151199 PMC6628105

[25] Ryan AS Nelson EB. Assessing the effect of docosahexaenoic acid on cognitive functions in healthy, preschool children: a randomized, placebo-controlled, double-blind study. Clin Pediatr. (2008) 47:355–62. doi: 10.1177/0009922807311730, PMID: 18180340

[26] Serhan CN Yacoubian S Yang R. Anti-inflammatory and proresolving lipid mediators. Annu Rev Pathol. (2008) 3:279–312. doi: 10.1146/annurev.pathmechdis.3.121806.151409, PMID: 18233953 PMC2739396

[27] Serhan CN Gupta SK Perretti M Godson C Brennan E Li Y. The atlas of inflammation resolution (AIR). Mol Asp Med. (2020) 74:100894. doi: 10.1016/j.mam.2020.100894, PMID: 32893032 PMC7733955

[28] Sun Y Chatterjee R Ronanki A Ye K. Circulating polyunsaturated fatty acids and COVID-19: a prospective cohort study and Mendelian randomization analysis. Front Med. (2022) 9:923746. doi: 10.3389/fmed.2022.923746, PMID: 35783629 PMC9243664

[29] Harris WS Tintle NL Sathyanarayanan SP Westra J. Association between blood N-3 fatty acid levels and the risk of coronavirus disease 2019 in the UK biobank. Am J Clin Nutr. (2023) 117:357–63. doi: 10.1016/j.ajcnut.2022.11.011, PMID: 36863828 PMC9972865

[30] Schuchardt JP Tintle N Westra J Harris WS. Estimation and predictors of the omega-3 index in the UK biobank. Br J Nutr. (2023) 130:312–22. doi: 10.1017/S0007114522003282, PMID: 36210531 PMC10277661

[31] Walker RE Jackson KH Tintle NL Shearer GC Bernasconi A Masson S. Predicting the effects of supplemental EPA and DHA on the omega-3 index. Am J Clin Nutr. (2019) 110:1034–40. doi: 10.1093/ajcn/nqz161, PMID: 31396625

[32] Zapata BR Müller JM Vásquez JE Ravera F Lago G Cañón E. Omega-3 index and clinical outcomes of severe COVID-19: preliminary results of a cross-sectional study. Int J Environ Res Public Health. (2021) 18:7722. doi: 10.3390/ijerph18157722 34360016 PMC8345773

[33] Mazidimoradi A Alemzadeh E Alemzadeh E Salehiniya H. The effect of polyunsaturated fatty acids on the severity and mortality of COVID patients: a systematic review. Life Sci. (2022) 299:120489. doi: 10.1016/j.lfs.2022.120489, PMID: 35358595 PMC8958853

[34] Nursyifa Fadiyah N Megawati G Erlangga Luftimas D. Potential of omega 3 supplementation for coronavirus disease 2019 (COVID-19): a scoping review. Int J Gen Med. (2022) 15:3915–22. doi: 10.2147/IJGM.S357460, PMID: 35431568 PMC9012318

[35] Doaei S Gholami S Rastgoo S Gholamalizadeh M Bourbour F Bagheri SE. The effect of omega-3 fatty acid supplementation on clinical and biochemical parameters of critically ill patients with COVID-19: a randomized clinical trial. J Transl Med. (2021) 19:128. doi: 10.1186/s12967-021-02795-5, PMID: 33781275 PMC8006115

[36] Mozaffarian D Lemaitre RN King IB Song X Huang H Sacks FM. Plasma phospholipid long-chain ω-3 fatty acids and total and cause-specific mortality in older adults: a cohort study. Ann Intern Med. (2013) 158:515–25. doi: 10.7326/0003-4819-158-7-201304020-00003, PMID: 23546563 PMC3698844

[37] Eide IA Jenssen T Hartmann A Diep LM Dahle DO Reisæter AV. The association between marine n-3 polyunsaturated fatty acid levels and survival after renal transplantation. Clin J Am Soc Nephrol. (2015) 10:1246–56. doi: 10.2215/CJN.11931214, PMID: 26063768 PMC4491303

[38] Di Profio E Risé P Orlandi L Zoia E Pinna C Sala A. Unsaturated fatty acids, omega-3 index and hospitalization in MISC. Prostaglandins Leukot Essent Fatty Acids. (2024) 202:102627. doi: 10.1016/j.plefa.2024.102627, PMID: 38964007

[39] Nobili V Bedogni G Alisi A Pietrobattista A Risé P Galli C. Docosahexaenoic acid supplementation decreases liver fat content in children with non-alcoholic fatty liver disease: double-blind randomised controlled clinical trial. Arch Dis Child. (2011) 96:350–3. doi: 10.1136/adc.2010.192401, PMID: 21233083

[40] CDC. Growth charts—2000 CDC growth charts—United States. Centers for Disease Control and Prevention (2022) Available online at: https://www.cdc.gov/growthcharts/cdc_charts.htm (Accessed January 15, 2024).

[41] CDC. Growth charts—CDC extended BMI-for-age growth charts. Centers for Disease Control and Prevention (2022) Available online at: https://www.cdc.gov/growthcharts/extended-bmi.htm (Accessed January 15, 2024).

[42] Simental-Mendía LE Rodríguez-Morán M Guerrero-Romero F. The product of fasting glucose and triglycerides as surrogate for identifying insulin resistance in apparently healthy subjects. Metab Syndr Relat Disord. (2008) 6:299–304. doi: 10.1089/met.2008.0034, PMID: 19067533

[43] Marangoni F Colombo C Galli C. A method for the direct evaluation of the fatty acid status in a drop of blood from a fingertip in humans: applicability to nutritional and epidemiological studies. Anal Biochem. (2004) 326:267–72. doi: 10.1016/j.ab.2003.12.016, PMID: 15003567

[44] Stark KD Aristizabal Henao JJ Metherel AH Pilote L. Translating plasma and whole blood fatty acid compositional data into the sum of eicosapentaenoic and docosahexaenoic acid in erythrocytes. Prostaglandins Leukot Essent Fatty Acids. (2016) 104:1–10. doi: 10.1016/j.plefa.2015.11.002, PMID: 26802936

[45] Natto ZS Yaghmoor W Alshaeri HK Van Dyke TE. Omega-3 fatty acids effects on inflammatory biomarkers and lipid profiles among diabetic and cardiovascular disease patients: a systematic review and Meta-analysis. Sci Rep. (2019) 9:18867. doi: 10.1038/s41598-019-54535-x, PMID: 31827125 PMC6906408

[46] Simopoulos AP. Omega-3 fatty acids in inflammation and autoimmune diseases. J Am Coll Nutr. (2002) 21:495–505. doi: 10.1080/07315724.2002.10719248, PMID: 12480795

[47] Rogero MM Leão M d C Santana TM Pimentel MV d MB Carlini GCG da Silveira TFF. Potential benefits and risks of omega-3 fatty acids supplementation to patients with COVID-19. Free Radic Biol Med. (2020) 156:190–9. doi: 10.1016/j.freeradbiomed.2020.07.005 32653511 PMC7350587

[48] Calder PC. Eicosanoids. Essays Biochem. (2020) 64:423–41. doi: 10.1042/EBC20190083, PMID: 32808658

[49] Donoghue V Schleicher GK Spruyt MGL Malan L Nel DG Calder PC. Four-oil intravenous lipid emulsion effect on plasma fatty acid composition, inflammatory markers and clinical outcomes in acutely ill patients: a randomised control trial (foil fact). Clin Nutr. (2019) 38:2583–91. doi: 10.1016/j.clnu.2018.12.010, PMID: 30638739

[50] Zaloga GP. Narrative review of n-3 polyunsaturated fatty acid supplementation upon immune functions, resolution molecules and lipid peroxidation. Nutrients. (2021) 13:662. doi: 10.3390/nu13020662, PMID: 33670710 PMC7922327

[51] Weill P Plissonneau C Legrand P Rioux V Thibault R. May omega-3 fatty acid dietary supplementation help reduce severe complications in Covid-19 patients? Biochimie. (2020) 179:275–80. doi: 10.1016/j.biochi.2020.09.003, PMID: 32920170 PMC7481803

[52] Gallo CG Fiorino S Posabella G Antonacci D Tropeano A Pausini E. The function of specialized pro-resolving endogenous lipid mediators, vitamins, and other micronutrients in the control of the inflammatory processes: possible role in patients with SARS-CoV-2 related infection. Prostaglandins Other Lipid Mediat. (2022) 159:106619. doi: 10.1016/j.prostaglandins.2022.106619, PMID: 35032665 PMC8752446

[53] Turner D Shah PS Steinhart AH Zlotkin S Griffiths AM. Maintenance of remission in inflammatory bowel disease using omega-3 fatty acids (fish oil): a systematic review and meta-analyses. Inflamm Bowel Dis. (2011) 17:336–45. doi: 10.1002/ibd.21374, PMID: 20564531

[54] Balas L Risé P Gandrath D Rovati G Bolego C Stellari F. Rapid Metabolization of Protectin D1 by β-oxidation of its polar head chain. J Med Chem. (2019) 62:9961–75. doi: 10.1021/acs.jmedchem.9b01463, PMID: 31626541

[55] World Health Organization C-reactive protein concentrations as a marker of inflammation or infection for interpreting biomarkers of micronutrient status. Vitamin and mineral nutrition information system. (2014) Available online at: https://www.who.int/publications/i/item/WHO-NMH-NHD-EPG-14.7 (Accessed March 21, 2025).

[56] World Health Organization WHO guideline on use of ferritin concentrations to assess iron status in individuals and populations. (2020). Available online at: https://www.who.int/publications/i/item/9789240000124 (Accessed March 21, 2025). 33909381

[57] Tishkowski K Gupta V. Erythrocyte Sedimentation Rate In: StatPearls. Treasure Island (FL): StatPearls Publishing (2024). Available at: https://www.ncbi.nlm.nih.gov/books/NBK557485/ (Accessed March 21, 2025). 32491417

[58] Guirao JJ Cabrera CM Jiménez N Rincón L Urra JM. High serum IL-6 values increase the risk of mortality and the severity of pneumonia in patients diagnosed with COVID-19. Mol Immunol. (2020) 128:64–8. doi: 10.1016/j.molimm.2020.10.006, PMID: 33075636 PMC7556792

[59] Gounden V Vashisht R Jialal I. Hypoalbuminemia. StatPearls. Treasure Island (FL): StatPearls Publishing (2024). Available at: https://www.ncbi.nlm.nih.gov/books/NBK526080/ (Accessed March 21, 2025). 30252336

[60] Townsend L Fogarty H Dyer A Martin-Loeches I Bannan C Nadarajan P. Prolonged elevation of D-dimer levels in convalescent COVID-19 patients is independent of the acute phase response. J Thromb Haemost. (2021) 19:1064–70. doi: 10.1111/jth.15267, PMID: 33587810 PMC8013297

[61] Boyarchuk O Perestiuk V Kosovska T Volianska L. Coagulation profile in hospitalized children with COVID-19: pediatric age dependency and its impact on long COVID development. Front Immunol. (2024) 15:410. doi: 10.3389/fimmu.2024.1363410, PMID: 38510249 PMC10950941

[62] National Heart, Lung, and Blood Institute. Expert panel on integrated guidelines for cardiovascular health and risk reduction in children and adolescents: summary report. Pediatrics. (2011) 128:S213–56. doi: 10.1542/peds.2009-2107C 22084329 PMC4536582

[63] Vieira-Ribeiro SA Fonseca PCA Andreoli CS Ribeiro AQ Hermsdorff HHM Pereira PF. The TyG index cutoff point and its association with body adiposity and lifestyle in children. J Pediatr. (2019) 95:217–23. doi: 10.1016/j.jped.2017.12.012, PMID: 29457996

[64] Calcaterra V Montalbano C de Silvestri A Pelizzo G Regalbuto C Paganelli V. Triglyceride glucose index as a surrogate measure of insulin sensitivity in a Caucasian pediatric population. J Clin Res Pediatr Endocrinol. (2019). doi: 10.4274/jcrpe.galenos.2019.2019.0024 31088046

