# Supplementary material for: Exploratory study of the effect of DHA supplementation on blood fatty acids and inflammatory markers in children with MIS-C
Source: Front Nutr. 2025 Jul 16;12:1597868. doi: 10.3389/fnut.2025.1597868 (PMC12307189; doi:10.3389/fnut.2025.1597868)
Supplement: Supplementary file 1 [file Table_1.docx]

**Supplementary materials:**

Supplementary Table 1: composition of DHA algae supplement.

Supplementary Table 2: Differences in baseline fatty acids profile between Group 1 and Group 2

Supplementary Table 3: Whole blood fatty acids profile in Group 1 and Group 2 at T0, T1 and T2 compared to reference ranges reported in the literature.

Suppl. Table 1: composition of DHA algae supplement

|  | 100 ml | 1 ml |
| --- | --- | --- |
| Energy | 451 kcal | 5 kcal |
| Fat | 50 g | 0.50 g |
| -Saturated fat | <1 g | <0.01 g |
| -Monounsaturated fat | 0 g | 0 g |
| -Polyunsaturated fat | 25 g | 0,25 g |
| Carbohydrates | 0 g | 0 g |
| -Sugar | 0 g | 0 g |
| Protein | 0 g | 0 g |
| Salt | 0 g | 0 g |
| DHA (from algae oil, titrated 50% in DHA) | 25000 mg | 250 mg |

Suppl. Table 2 Differences in baseline fatty acids profile between Group 1 and Group 2

|  | **Group 1 T0 (DHA)** | **Group 2 T0 (NO DHA)** | **P value** |
| --- | --- | --- | --- |
| *Fatty acids profile [% w/w(SD)]* | | | |
| **Palmitic acid** | 27.92(1.66) | 29.43(2.45) | 0.058 |
| **Stearic acid** | 10.15(1.09) | 10.62(1.57) | 0.342 |
| **Arachidic acid** | 0.41(0.10) | 0.39(0.12) | 0.633 |
| **Behenic acid** | 1.04(0.22) | 1.06(0.21) | 0.795 |
| **Lignoceric acid** | 1.72(0.55) | 1.67(0.44) | 0.775 |
| **Palmitoleic acid** | 3.14(0.83) | 2.77(1.21) | 0.344 |
| **Oleic acid** | 27.07(3.39) | 27.67(4.27) | 0.672 |
| **7-Octadecenoic acid** | 1.65(0.28) | 1.63(0.32) | 0.855 |
| **Eicosenoic acid** | 0.20(0.13) | 0.19(0.08) | 0.986 |
| **Eruric acid** | 0.08(0.03) | 0.07(0.05) | 0.712 |
| **Nervonic acid** | 2.11(0.58) | 2.00(0.44) | 0.579 |
| **Eicosatrienoic acid** | 0.17(0.10) | 0.15(0.09) | 0.630 |
| **Linoleic acid (LA)** | 12.57(2.22) | 12.70(2.27) | 0.876 |
| **Gamma-linolenic acid** | 0.68(0.41) | 0.33(0.31) | **0.014** |
| **Dihomogammalinolenic acid** | 1.13(0.30) | 0.94(0.32) | 0.111 |
| **Arachidonic acid (AA)** | 6.60(1.21) | 5.31(1.84) | **0.031** |
| **Adrenic acid** | 0.84(0.26) | 0.65(0.29) | 0.070 |
| **Docosapentaenoic acid (DPA) n-6** | 0.36(0.08) | 0.33(0.16) | 0.431 |
| **Alfa-linolenic acid (ALA)** | 0.20(0.09) | 0.17(0.06) | 0.272 |
| **Eicosapentaenoic acid (EPA)** | 0.32(0.09) | 0.41(0.11) | **0.021** |
| **Docosapentaenoic acid (DPA)** | 0.48(0.10) | 0.41(0.22) | 0.250 |
| **Docosahexaenoic acid (DHA)** | 1.19(0.25) | 1.09(0.51) | 0.530 |
| **Total saturateds FAs** | 41.23(2.42) | 43.17(3.34) | 0.080 |
| **Total Monounsaturated FAs** | 34.24(3.61) | 34.35(4.64) | 0.945 |
| **Total Polyunsaturated FAs** | 24.52(2.75) | 22.48(4.60) | 0.151 |

Suppl. Table 3 Whole blood fatty acids profile (% w/w±SD) in Group 1 and Group 2 at T0, T1 and T2 compared to reference ranges reported in the literature.

| Group/Author | Group 1 at T0, T1 and T2 | | | Group 2 at T0, T1 and T2 | | | Risé [17] | Crippa [18] | Bonafini[16] | Van der Wurff [19] | Ryan [20] |
| --- | --- | --- | --- | --- | --- | --- | --- | --- | --- | --- | --- |
| Age (year) | 2-18 years | | | 2-18 years | | | <9 | 7-14 | 7-9 | 13-15 | 4 |
| % LA | 12,57 ±2,22 | 14,56±1,58 | 17,71±2,82 | 12,70±2,27 | 17,92±2,66 | 19,29±1,94 | 17,6±1,92 | 22,54±2,45 | 19,9±2,32 |  |  |
| % AA | 6,60±1,21 | 9,05±1,79 | 9,29±2,20 | 5,31±1,84 | 7,35±1,38 | 8,30±1,55 | 8,33±1,04 | 10,10±0,92 | 12,21±1,67 | 11,01±11,33 | 7,50±1,89 |
| % ALA | 0,20±0,09 | 0,29±0,16 | 0,21±0,08 | 0,17±0,06 | 0,36±0,16 | 0,20±0,06 | 0,15±0,05 |  | 0,16±0,08 |  |  |
| % EPA | 0,32±0,09 | 0,46±0,10 | 0,41±0,08 | 0,41±0,11 | 0,28±0,10 | 0,33±0,09 | 0,23±0,08 | 1,13±0,45 | 0,30±0,17 | 0,34±0,42 | 0,30±0,39 |
| % DHA | 1,19±0,25 | 2,67±0,78 | 2,37±0,87 | 1,09±0,51 | 1,44±0,47 | 1,70±0,43 | 1,40±0,37 | 1,93±0,53 | 2,92±0,76 | 2,49±2,63 | 1,00±0,34 |
